# Relationship between anti-Müllerian hormone concentration and antral follicle count in Colombian creole cows of the Chino Santandereano breed

**DOI:** 10.5455/javar.2025.l931

**Published:** 2025-07-11

**Authors:** Héctor Javier Narváez, Diego Armando Vega Borda, Esneyder Rugeles Ballesteros, Deicy Villalba Rey, Ricardo Lopes Dias da Costa

**Affiliations:** 1 Facultad de Ciencias Exactas, Naturales y Agropecuarias, Universidad de Santander, Bucaramanga, Colombia.; 2 Facultad de Ciencias, Universidad del Tolima, Ibagué, Colombia.; 3 Instituto de Zootecnia, Nova Odessa, Brazil.

**Keywords:** Creole cattle, estrous cycle, follicle, hormones, ovarian reserve.

## Abstract

**Objective:**

This study was to evaluate the relationship between serum anti-Müllerian hormone (AMH) concentration and antral follicle count (AFC) during the estrous cycle of Colombian Creole cows of the Chino Santandereano breed.

**Materials and Methods:**

Ten non-lactating, non-pregnant, multiparous cows of the Chino Santandereano breed (*Bos taurus taurus* adapted), aged between 3 and 7 years, with a body condition of 3.0 ± 0.4 and with normal reproductive tracts at the structural and functional level, were selected and used. For the synchronization of estrus and ovulation, an intravaginal progesterone release device plus 2 mg of estradiol benzoate was applied for 8 days. On day 8, 150 μg of cloprostenol sodium + 300 IU of equine chorionic gonadotropin + 1 mg of estradiol cypionate was administered. This protocol was performed to determine the antral follicular count and to quantify serum AMH levels every 5 days during the estrous cycle.

**Results:**

The mean serum AMH concentration and AFC were 725 ± 2.7 pg/ml and 43.4 ± 3.5, respectively. A high correlation was observed between AMH and AFC of *r* = 0.041; *p* < 0.0001.

**Conclusion:**

The results showed that in Creole cows of the Chino Santandereano breed, there is a high correlation between circulating levels of AMH and ovarian reserve. This mechanism can be used as an endocrine biomarker of the follicular population.

## Introduction

Exposure to a protracted period of natural selection has led to the adaptation of Colombian Creole cattle to a broad array of geographic and environmental conditions. These animals have developed characteristics that enable them to survive food shortages, exhibit high productivity and reproductive efficiency, and demonstrate high resistance to diseases and parasites, high tolerance to adverse climatic conditions, and greater longevity [1]. These characteristics allowed them to survive over time; however, very little is known about the reproductive physiology of the Creole breeds of Colombia, particularly the Chino Santandereano breed (*Bos taurus taurus* adapted), currently cataloged as at risk of disappearance, with an effective population of 374 animals [2].

Anti-Müllerian hormone (AMH) is a 140 kDa dimeric glycoprotein belonging to the transforming growth factor beta (TGF ß) superfamily [3]. In bovine females, it is secreted by granulosa cells of primary, secondary, pre-antral, and small antral follicles, decreasing its expression during the final phase of follicular development [4]. This expression pattern indicates that AMH plays an important role in the regulation of the number of growing follicles, as well as in the mechanism of follicular selection and ovulation [4].

The study classified the plasma concentration of AMH into 3 groups: low concentration (10–140 pg/ml), intermediate concentration (141–450 pg/ml), and high concentration (451–3,198 pg/ml) [5]. Nonetheless, there is evidence that AMH concentration varies minimally during the bovine estrous cycle. However, the variation in circulating hormone levels between individuals is significant [6].

Circulating levels of AMH have been related to the size of the ovarian reserve in cattle, which is considered a marker of follicular population because AMH in females is primarily produced by the granulosa cells of healthy and growing ovarian follicles [3, 7, 8]. Studies suggest that AMH has a high influence on the mechanisms of establishment and maintenance of the dominant follicle after follicle selection [9].

In addition, there is a strong correlation between plasma AMH concentrations and antral follicle count (AFC), which means that low AMH concentrations coincide with a lower number of antral follicles [10]. The authors collected blood samples daily for a period of 6 to 9 days prior to ovulation in taurine females. Their observations revealed that the mean concentration of the hormone was 2 to 6 times higher in animals with high follicular counts and intermediate follicular counts than in females with low follicular counts. This finding indicates a positive correlation with the follicular population. Ireland et al. [8] developed a classification system for antral follicles, which was based on the number of follicles present. The classification system included three categories: low follicle count (≤ 15 follicles), intermediate follicle count (16–24 follicles), and high follicle count (≥ 25 follicles).

Several studies have indicated no or limited association between AMH concentration and ovarian reserve with fertility in Holstein (*B. taurus taurus*) females [11–13]. These findings indicate a greater effect of environmental and genetic factors on the size of the ovarian reserve and circulating levels of AMH.

For this reason, the present study aimed to determine the relationship between AMH concentration and AFC in Creole cows of the Chino Santandereano breed (*B. taurus taurus* adapted) during the estrous cycle.

## Materials and Methods

### Ethical approval

The present study was developed in accordance with the laws and regulations of Colombia, established by resolution 8,430 of 1993, which contemplates biomedical research with animals. The study was approved by the Ethics and Bioethics Committee of the University of Santander (minute No. 27 of December 9, 2020).

### Selection and management of animals

A total of ten non-lactating, non-pregnant Chino Santandereano (*B. taurus taurus* adapted) subjects of Creole breed, aged between three and seven years, with a body condition score of 3.0 ± 0.4 on a scale of 1 to 5 points, and with a normal reproductive tract at a structural and functional level, were selected and used in the study. The cows were selected from a nucleus located in the department of Santander (Colombia), which was free of brucellosis and tuberculosis. The cows were vaccinated against foot and mouth disease, leptospirosis, bovine viral diarrhea, and infectious bovine rhinotracheitis. The study was carried out under tropical conditions with an average temperature of 22°C throughout the experimental period, with *Brachiaria decumbens* pasture, mineral supplementation, and water *ad libitum*.

All the manipulations performed on the animals: blood collection and ultrasound scans. They were carried out in cattle handling pens, an installation that allows the animals to be safely contained, restricting their movements by restraint in the lateral region of the neck and the line of the paralumbar fossa, preserving animal welfare.

### Estrus and ovulation synchronization protocol

For the synchronization of estrus and ovulation, a 1 gm intravaginal progesterone release device (Sincrogest®, Ouro Fino, Brazil) was applied for 8 days, plus the application of 2 mg of estradiol benzoate (Sincrodiol®, Ouro Fino, Brazil). Once the P4 device was removed, the application of 150 μg cloprostenol sodium (Sincrocio®, Ouro Fino, Brazil), 300 IU of equine chorionic gonadotropin (Sincro eCG®, Ouro Fino, Brazil), plus the administration of 1 mg of estradiol cypionate (SincroCP®, Ouro Fino, Brazil) was performed. Ovulation occurred 48 h after removal of the progesterone device. This protocol was performed to determine the antral follicular count and to quantify serum AMH levels every 5 days during the estrous cycle.

### Antral follicular count

The ultrasonographic examination was initiated on the day of ovulation (D0) and performed every 5 days throughout the estrous cycle (D5, D10, D15, and D20). For the quantification of the follicular population, a DP 30 Vet model ultrasound scanner (Mindray Vet, China), equipped with a linear transducer with a frequency of 7.5 MHz, was used. According to the number of antral follicles, the females were classified into three groups: low AFC (≤ 15), intermediate AFC (16 to 24), and high AFC (≥ 25). These criteria were in accordance with Ireland et al. [8].

### Quantification of serum AMH concentration

Once the ultrasonographic evaluations were performed on each of the cows, they were subjected to quantification of the serum concentration of AMH by collecting 6 ml of blood from the coccygeal vein in EDTA-free vacutainer tubes. The samples were centrifuged at 3,000 G for 20 min, after which the serum was conditioned in 2 ml cryotubes for storage at –20°C until analysis. The analysis of serum AMH concentration was determined with the aid of a commercial ELISA kit (Bovine AMH ELISA AL-114, AnshLabs^®^, Webster, TX, U.S.A.), the concentration was expressed in pg/ml, and the inter-assay coefficient of variation was 0.28 to 3.15. Samples were thawed and homogenized by centrifugation for 5 min at 6,000 G. Before starting the procedure, all calibrators, controls, samples, and hormone assay buffers were prepared using the methodology described in the kit.

Samples were collected every 5 days from ovulation (D0) until day 20 of the estrous cycle. Serum AMH concentration was classified into low (10–140 pg/ml), intermediate (141–450 pg/ml), and high (451–3,198 pg/ml) [5].

### Statistical analysis

The comparison of follicular population and plasma concentration of AMH was analyzed according to the analysis of variance method. For comparisons between serum AMH concentration and the number of antral follicles visualized, the individual difference probability test was used.

Calculations were performed with the SPSS statistical program (IBM Corp., 2020; IBM SPSS Statistics for Windows, Version 27.0) In the statistical tests, a value of *p* ≤ 0.05 was considered as a criterion for acceptance of statistically significant effects.

## Results

**Figure 1.**
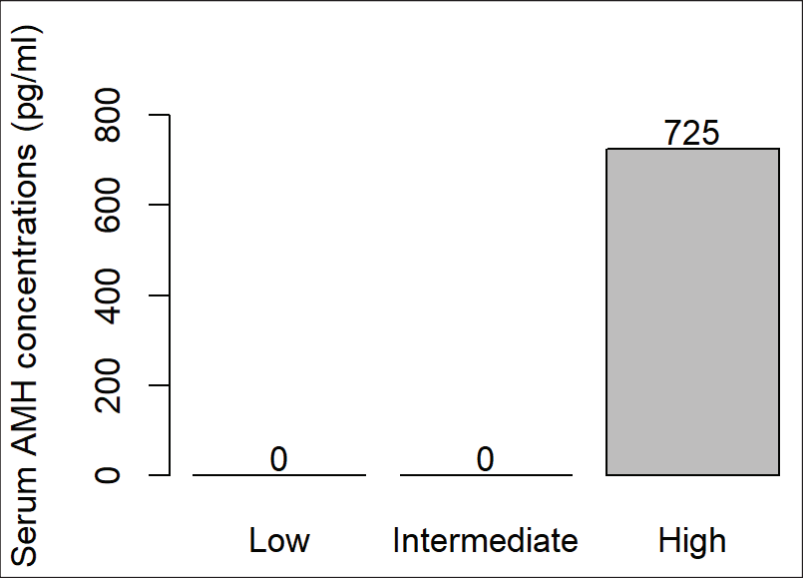
Classification of serum AMH levels (pg/ml) in creole cows of Chino Santandereano breed.

In the present study, the average serum concentration of AMH per cow (±SEM) was 725 ± 2.7 pg/ml, with a range of 514.6 to 943.3 pg/ml per animal (Fig. 1). All females under study had high serum levels of AMH.

**Figure 2.**
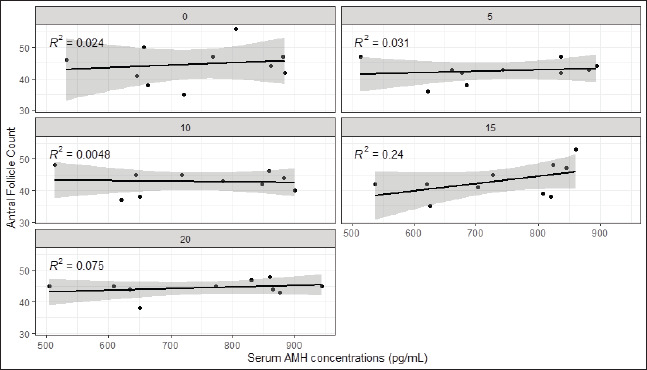
Correlation between serum AMH concentration and antral follicular count on days 0, 5, 10, 15, and 20 of the estrous cycle in creole cows of the Chino Santandereano breed.

The concentrations of AMH that were present in the blood during the days 0, 5, 10, 15, and 20 of the estrous cycle were 741.9 ± 1.5, 735.6 ± 2.9, 741.9 ± 31, 737.4 ± 2.7, and 754.3 ± 2.5 pg/ml, respectively. The data are shown in Figure 2.

**Figure 3.**
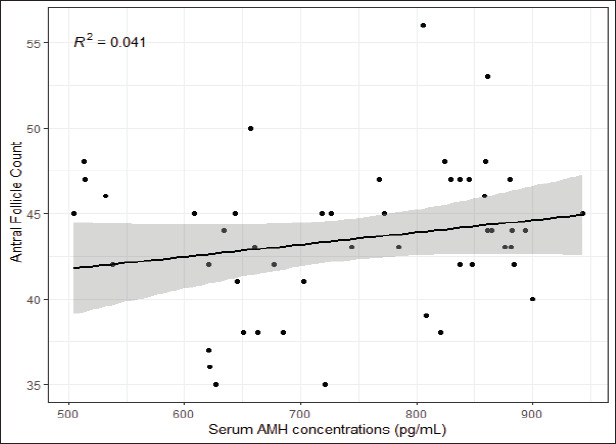
Classification of serum AMH levels (pg/ml) in creole cows of Chino Santandereano breed.

The average results yielded a positive correlation (*r* = 0.041; *p* < 0.0001) between serum AMH concentration and AFC (Fig. 3).

**Figure 4.**
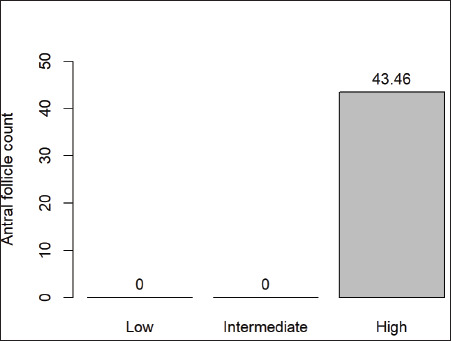
Classification of the antral follicular population in creole cows of the Chino Santandereano breed.

The average AFC was 43.4 ± 3.5, and the individual AFC ranged from 35 to 56 follicles. These results indicate that the Chino Santandereano females studied had a high follicular population (Fig. 4).

## Discussion

This is the inaugural study to be conducted in Colombian Creole cattle and the Chino Santandereano breed (*B. taurus taurus* adapted), with the objective of ascertaining the relationship between AMH and AFC. The present study offers novel insights into the reproductive physiology of this significant Colombian animal genetic resource.

In our study, a linear association between serum AMH levels and AFC was observed (*r* = 0.041; *p* < 0.0001). These results are consistent with those described by Batista et al. [14] in Nelore (*Bos taurus indicus*) and Holstein (*B. taurus taurus*) females. According to Ireland et al. [6], higher circulating concentrations of AMH have been observed in animals with intermediate and/or high antral follicular counts compared to those with low follicular counts. More recent studies obtained similar results, demonstrating a strong association between AMH and AFC (*r* = 0.613) [15]. Another study obtained a moderate correlation (*r* = 0.34; *p* < 0.001) between serum AMH concentration and AFC in *B. taurus taurus* females (Holstein, Red Holstein, Brown Swiss, and Montbéliard) [16].

The present study examined the repeatability of serum AMH levels in Chino Santandereano cows. The quantification of these levels was performed on different days of the same estrous cycle. The results demonstrated a high degree of repeatability (0.87). There is evidence that a single measurement or several measurements during a single estrous cycle or multiple cycles present a high correlation (*r* = 0.97) [6, 17]. The study encompasses animals that exhibit estrus naturally or through hormonal synchronization [18].

The results presented in the current study in Creole females of the Chino Santandereano breed are consistent with those previously reported by Gobikrushanth et al. [19] in Holstein cows, where they presented a repeatability of 0.73. As indicated by the findings of other studies, there is a high degree of repeatability in Holstein cows subjected to superovulation [20]. Additionally, the research indicates a repeatability of 0.90 in *B. taurus taurus* cows (Holstein, Jersey, and Holstein × Jersey) during the lactation period [5].

Another concomitant aspect is the repeatability of the AFC; in the Creole cows of the current study, a value considered high (0.82) was presented. According to Ireland et al. [6], the number of antral follicles recruited per follicular wave is highly variable (range 8–54) among individuals. Nevertheless, it is considered a characteristic of high repeatability (0.95) during the entire estrous cycle or consecutive estrous cycles of the same animal [21].

Notwithstanding the findings outlined earlier, the results of the present study differ from those previously documented in the extant literature. This discrepancy is further accentuated by the findings of Gobikrushanth et al. [19], who reported a repeatability of 0.37 in lactating Holstein cows. Subsequent studies confirmed that AFC repeatability is independent of age, genetic group, environment, lactation period, and time interval between AFC assessments [8, 11, 21, 22].

The antral follicular count of the current study was higher than the results obtained by Vega et al. [23] in Creole cows of the same genetic group. These authors visualized a follicular population of 12.1 ± 0.5 structures. However, a previous study carried out in another genetic group of Colombian Creole cows (Blanco Orejinegro) presented an antral follicular population of 30.4 ± 0.6 [24].

Studies found that in Holstein heifers (*B. taurus taurus*), the AFC presented a variation from 18 to 110 structures [25] or from 3 to 36 [11], and in cows of the same genetic group in the lactation period, the AFC ranged from 4 to 61 [22]. In Angus (*B. taurus taurus*) heifers, AFC ranged from 7 to 54 [26].

In lactating Nelore cows (*B. taurus indicus*), the observed AFC ranged from 21 to 51 [27]; another study reported values from 2 to 50 [28]. In more recent results, in cows of the same genetic group and productive status, the AFC was from 11.3 to 49.5 [29].

Some studies have correlated AFC with fertility in cattle. According to Succu et al. [11], fertility in Holstein females is enhanced by a high follicular population. Researchers hypothesized that dairy cows of the same genetic group with low AFC could be associated with reduced fertility [22]. In the case of Holstein heifers, it has been observed that animals with high AFC exhibit diminished reproductive performance and a reduced productive lifespan [21]. In *B. taurus indicus* (Nelore) heifers, it was determined that animals with low AFC present higher regulation of genes related to epigenetic modulation, meiotic control, follicular growth, cellular maintenance, and a better response to cellular stress [27].

## Conclusion

The results showed that in Colombian Creole cows of the Chino Santandereano breed, there is a high association between circulating levels of AMH and ovarian reserve. This mechanism serves as an endocrine biomarker for the follicular population. However, the interplay between AFC, AMH, and reproduction remains to be fully elucidated, hindering the development of reliable methods for predicting fertility in bovine females.

## List of abbreviations

°C, Degree Celsius

AFC, Antral follicle count

AMH, AntiMüllerian hormone

CP, Estradiol cypionate

D, Day

eCG, Equine chorionic gonadotropin

gm, grams

h, hours

IU, International units

mg, milligrams

pg/ml, picograms per milliliter

TGF ß, Growth factor beta

μg, microgram.
